# Population structure, age distribution and sex composition of long-tailed gorals in Jangsudae, Seoraksan National Park, Republic of Korea

**DOI:** 10.3897/BDJ.14.e178454

**Published:** 2026-03-12

**Authors:** Sangjin Lim, Maniram Banjade, Ki-Yoon Kim, Anya Lim, Yungchul Park

**Affiliations:** 1 College of Forest and Environmental Sciences, Kangwon National University, Chuncheon, Republic of Korea College of Forest and Environmental Sciences, Kangwon National University Chuncheon Republic of Korea; 2 Wildlife Ecology Institute, ECOEM Inc., Wonju-si, Republic of Korea Wildlife Ecology Institute, ECOEM Inc. Wonju-si Republic of Korea; 3 College of Veterinary Medicine, Chungbuk National University, Cheongju, Republic of Korea College of Veterinary Medicine, Chungbuk National University Cheongju Republic of Korea; 4 Endangered Species Restoration Center, National Institute of Ecology, Yangyang, Republic of Korea Endangered Species Restoration Center, National Institute of Ecology Yangyang Republic of Korea

**Keywords:** conservation, endangered, long-tailed goral, population structure, sexual distribution

## Abstract

The long-tailed goral (*Naemorhedus caudatus*) is an endangered species in Northeast Asia and is threatened by habitat loss, fragmentation and human activities. This study investigated the population structure, age distribution and sex ratio of long-tailed gorals in the Jangsudae area of Seoraksan National Park, Republic of Korea, using camera traps and morphological identification methods. A total of 895 photos yielded data on 29 individuals, including 23 individuals aged > 1 year and 6 offspring, resulting in an age ratio of 3.8:1 (adult:juvenile). The population had a female biased sex ratio (0.3:1; male:female) in individuals >1 year old and no male in adult (3-10 years) and subadult (1–3 years) age groups. The age structure showed high number of older adults (i.e. 14) indicating a mature population, but limited number of young individuals suggesting survival challenges. The data on population structure and demographics of long-tailed gorals will be used as a basis for developing effective conservation and management plans for this endangered species. By showing population trends and reproductive dynamics, this study will provide insights for targeted conservation and long-term population sustainability.

## Introduction

The long-tailed goral (*Naemorhedus caudatus*) is an endangered ungulate species distributed across mountain habitats in Northeast Asia. Listed as vulnerable by the International Union for Conservation of Nature, its population has significantly declined owing to habitat loss, fragmentation and human activities (Bragina et al. 2020, Lim et al. 2025). In the Republic of Korea, where the long-tailed goral is designated as a natural monument and an endangered species (Lim et al. 2025a), its population is < 1,000 (Song et al. 2017). To develop an effective conservation strategy, it is crucial to understand the population dynamics and structure of this species.

Seoraksan National Park, located in the middle of the Baekdu-daegan mountain range, is one of the four largest goral habitats in the Republic of Korea. The Park’s rugged terrain and diverse ecosystems provide ideal conditions for gorals, supporting a population of over 100 individuals (Song et al. 2017). As a source population for the surrounding Baekdu-daegan region, the Seoraksan population plays a vital role in the conservation of this species. However, detailed information on the population structure and demographics within a specific area of the Park is limited (Kim et al. 2020).

Traditional methods for studying goral populations, such as visual survey and faecal analysis are limited by low detectability, observer bias and difficulty in accurately inferring true abundance in rugged goral habitats (Myslenkov and Voloshina (1989), Cho et al. (2015a), Kim et al. (2018)). In recent years, camera trapping has emerged as a valuable tool for monitoring wildlife populations, offering improved insights into population dynamics and behaviour Banjade et al. 2021, Cordier et al. 2022, Fisher 2023, Banjade et al. 2024). Specifically, studies utilising camera trapping have advanced our understanding of goral populations in various regions Zaumyslova and Bondarchuk 2015, Park et al. 2018, Kim et al. 2020). These studies have provided valuable insights into goral ecology and behaviour; however, further research across different habitats within their range is necessary.

This study focused on the Jangsudae area of Seoraksan National Park, where the goral population is high (Lim et al. 2023). While previous studies have assessed the overall Seoraksan goral population, this research addresses a critical knowledge gap by providing the first detailed demographic analysis of a specific habitat area within the Park. Using camera trapping and morphological identification keys, we aimed to study the population structure, age distribution and sex ratio of long-tailed gorals in this critical habitat. Our goal was to provide critical insights into the current status of endangered species and baseline data for targeted conservation plans.

## Materials and Methods

**Study area**

Jangsudae, located in Seoraksan National Park in Republic of Korea, is renowned for its rough mountains and diverse wildlife. The landscape has an average slope of 25.3°, an elevation of 883.6 m and an aspect of 194.4° (Lim et al. 2023). The climate is temperate, with an annual mean temperature of 3.05°C and average precipitation of 1537.4 mm. The forest in this region is primarily temperate, with a mix of deciduous and coniferous trees (Shin et al. 2016). This region is home to 10 medium- and large-sized mammals, including three endangered species: the yellow-throated marten (*Martes flavigula*), leopard cat (*Prionailurus bengalensis*) and goral (*Naemorhedus caudatus*) (Choi et al. 2020).

**Camera trapping**

**Figure 1.**
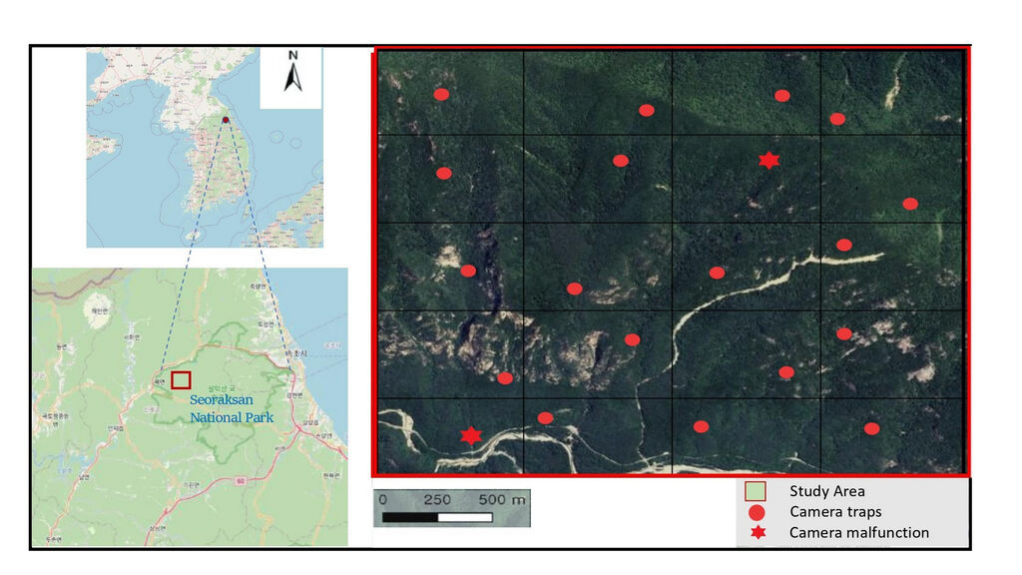
Geographical location of study sites and camera trap placements in Jangsudae, Seoraksan National Park, South Korea.

Data were collected from 7 July to 18 October 2016, in the Jangsudae region, encompassing a survey area of 5.76 km^2^ (2.4 km × 2.4 km). A total of 20 infrared cameras (Moultrie’s M-990i; PRADCO Outdoor Brands, Birmingham, AL, USA and SPYPOINT Force; Skypoint, Surfers Paradise, Australia) were deployed at 300–500 m intervals (Fig. 1). The cameras were positioned at an average height of 0.7–1.2 m above the ground to capture images of species, with trapping locations chosen, based on the signs of animal activity. Each camera was set to operate for 24 h per day, capturing three consecutive photographs at 1-min intervals, like the previous setup for goral counting (Kim et al. 2020). The cameras were inspected once a month to replace batteries and memory cards. The collected raw data were deposited at https://doi.org/10.5281/zenodo.17578718. 

**Data analysis**

**Individual identification**

We followed the methodology outlined by Kim et al. (2020) for identifying individual gorals. This approach uses morphological keys, including horn shapes, the proportion of rings on the horns, facial colour patterns and unique features (e.g. torn ear). Additional characteristics, such as body and head colour, tail length and shape also helped identify the individuals. Adults and offspring were distinguished, based on body size, horn maturity and the appearance of a discernible ring. To avoid double-counting, we used measures such as temporal gap analysis (carefully examining images taken within short intervals) and morphological cross-verification (manually reviewing individuals with identical identification codes for unique morphological markers) (Nakashima et al. 2022, Liu et al. 2024).

**Age identification**

The age of individuals was assessed using the methods described by Kim et al. (2020), based on the work of Myslenkov and Voloshina (1989). This method primarily relies on the proportion of horn rings to total horn length, with specific criteria for different age categories ranging from 1 to > 10 years. The surface of the horns is covered by skin at the beginning of its growth period and such horns are called newborn velvet and the horns that have been skeletonised later are called horns (Sokolov 1953). For individuals under one year old (i.e. those lacking visible horns or horn rings), maternal association was employed to assign offspring to their mothers, based on photographic proximity and behavioural cues. Adults were categorised into three groups according to the proportion of the horn covered by the rings. Subadults (1–3 years old) have ring proportions less than 20% of the horn length. The ring proportions varied from 21-50% in individuals aged 3–10 years, whereas it exceeded 50% in those aged 10 years and above (Myslenkov and Voloshina 1989, kim et al. 2020)

**Sex identification**

**Figure 2.**
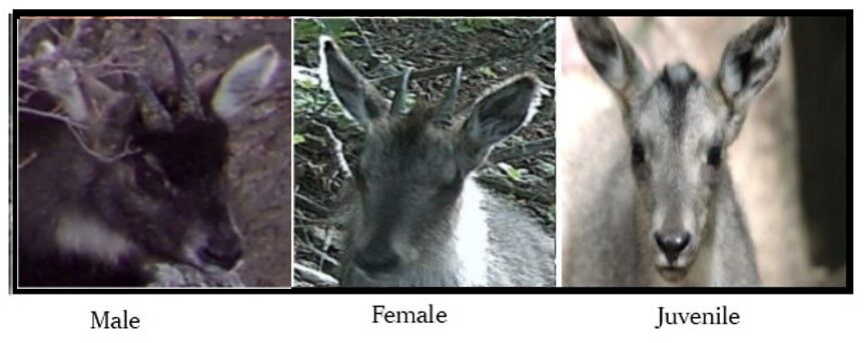
Distinct facial appearances and colour variations observed among males, females and juveniles at the study site. These differences may indicate sexual dimorphism and age-related developmental changes, providing potential cues for individual identification.

Sex determination was conducted following the criteria established by Kim et al. (2020), consistent with the method used by Myslenkov and Voloshina (1989) for long-tailed gorals in Russia. This approach is based on facial colour patterns (Kim 2017), with specific distinctions between males and females in the proportion and distribution of black colouration on the face (Fig. 2). Males have white hair close to the nose line and have a relatively dark-coloured face. The two horns are smaller and look parallel in females, whereas they are larger and orientated in a V shape in males (Myslenkov and Voloshina 1989, Park et al. 2018). In addition, male horns are severely bent backwards, the base looks thicker and the rings on the horns are much clearer than those of females.

## Results

A total of 895 photographs of long-tailed gorals were captured by 18 cameras (two of which malfunctioned) installed in the Jangsudae region of Seoraksan National Park. Based on morphological characteristics, only 660 photos were assigned to 29 individually identified gorals. However, individuals < 1 year old were identified by association with their mothers because of limited morphological identification. Of the 29 identified individuals, 12 (41%) were photographed only once, seven (24%) were captured twice and 10 (35%) were photographed three or more times. The high proportion of single captures suggests many individuals may have gone undetected, indicating the true local population size is likely larger than our detected sample.

The population comprised 23 individuals aged > 1 year and 6 offspring aged < 1 year, resulting in an age ratio of 3.8:1. However, one of the 23 individuals could not be classified by age or sex (Table 1). Amongst the 29 individuals, 14 were classified as older adults (≥ 10 years), four as adults (3–10 years), four as subadults (1–3 years) and six as offspring (< 1 year). In the older adult group (≥ 10 years), the sex ratio was initially calculated as equal, with seven males and seven females. However, only female individuals were observed in the 3–10-year adult and 1–3-year subadult group, resulting in a sex ratio of 0:4 in each group. For old adults aged > 10 years, the estimated error rate for misidentification of females as males was 12.5% (Kim et al. 2020). Consequently, one individual initially counted as male was reclassified as female, resulting in a final sex composition of six males and eight females in this age group. The corrected overall sex ratio for gorals aged > one years, excluding offspring and unidentified individuals was 0.37:1. This was further divided down to 0:4 for subadults, 0:4 for adults and 0.75:1 for older adults.

## Discussion

This study investigated the population structure and individual identification of long-tailed gorals in the Jangsudae area of Seoraksan National Park, Republic of Korea. A total of 29 individuals were identified from 895 photographs. The population comprised 23 individuals aged > 1 year and six offspring (< 1 year), resulting in an age ratio of 3.8:1. The sex ratio of gorals > 1 year was 0.3:1, indicating a female-biased population. These findings contribute to understanding the population dynamics of long-tailed goral population in Seoraksan National Park, one of the largest wild goral populations in Republic of Korea.

Camera traps are effective tools for individual identification and wildlife population assessments (Karanth and Nichols 2011, Banjade et al. 2023). Photographic identification of 29 long-tailed gorals in the Jangsudae area of Seoraksan National Park was conducted using a methodology similar to that conducted in the Osaek Region of Republic of Korea (Kim et al. 2020). However, fewer individuals were identified in Jangsudae than in Osaek, possibly owing to the differences in survey areas. Our study area spanned 5.76 km^2^, whereas that in the Osaek Region was 9.6 km^2^, leading to relatively more individuals being observed in Osaek. Such differences in individual counts related to survey area sizes have been observed in other mountain ungulates globally. For example, studies on Alpine ibex (*Capra ibex*) in the European Alps have found that smaller, isolated study sites had fewer individuals because of resources and habitat limitations (Brambilla et al. 2020). Similarly, Himalayan tahr *(Hemitragus jemlahicus*) in Nepal have varying population sizes depending on the area of suitable habitat surveyed, with smaller areas having fewer individuals (Shrestha 2007). This disparity highlights the importance of considering survey area size when comparing population estimates across regions.

The higher goral numbers in the Osaek area might also be due to its ecological characteristics. Osaek has a wider elevation range from Daecheong-bong, the highest peak in Seoraksan National Park, to Osaek Village. This elevation gradient provides preferred habitats, such as high altitude rocky terrain (Cho et al. 2015b). Jangsudae with an average elevation of 883.6 m has less topographical diversity and fewer optimal habitats (Lim et al. 2023). Osaek also has more varied topography and richer vegetation which might support more food resources and connectivity to adjacent habitats. These results show the importance of habitat quality and resource availability in shaping goral population. This also emphasises the need for comprehensive ecological analysis to understand distribution pattern and provide information for conservation strategy that address the fine scale environmental factors affecting mountain ungulate population.

The age structure of long-tailed goral populations provides important insights into their health, reproductive potential and growth capacity. The age ratio in this study was 3.8:1 (23 individuals > 1 year to 6 offspring) indicates a mature population, with nearly half (48%) classified as older adults (>10 years). Our study observed a similar age ratio of long-tailed goral reported in the Osek Region, where the ratio was 3.3:1 (43 adult individuals to 13 offspring), with 45% of older adults, aged more than 10 years (Kim et al. 2020) However, this ratio was lower than that reported in a separate, large-scale study conducted in Odaesan National Park by a different study (kim et al. 2020). The similarity in age structure between these regions implies that long-tailed goral populations in protected areas may share common demographic patterns, possibly due to comparable environmental conditions and reproductive success rates (Lim 2019). However, the low number of subadult and young individuals suggests a limited survival rate, most likely due to environmental constraints or catastrophic weather events. Previous research has shown that high winter mortality disproportionately impacts yearlings and pregnant females, as evidenced by the Wangpi River Basin, where 89.04% of documented deaths were amongst these susceptible groups (Park and Hong 2021). Similarly, in 2010, several gorals were found dead in Gangwon-do Province, coinciding with the second-highest snow cover depth in a decade (Park et al. 2018). These intense conditions most likely resulted in a high mortality rate amongst young and subadult individuals, leaving only mature adults capable of enduring such hard winters. Since our study was conducted in 2016, the impact of this demographic bottleneck may still be evident, contributing to the high proportion of older adults observed in our study area. Further investigation into this skewed age distribution is required for understanding population dynamics and designing conservation strategies that address both age and reproductive dynamics to ensure the long-term viability of this endangered species.

The sex ratio is particularly intriguing, with a strong female bias in the overall population and a complete absence of males in the 3–10 year and 2–3 year age groups. Female-biased sex ratios have been observed in long-tailed gorals in Republic of Korea and Russia (Myslenkov and Voloshina 1989, Kim et al. 2020) and in Himalayan grey gorals in Pakistan (Abbas et al. 2012). Age and sex skewed distributions may be owing to environmental pressures that differentially affect males. Seasonal climatic variability can increase energy demand and limit foraging conditions, disproportionately affecting young males because of their relatively high exploratory behaviour and their peripheral social positions, which make them more vulnerable to mortality (Lee et al. 2019, Park and Hong 2021). Additionally, males’ larger home ranges (Lim et al. 2025) may expose them to greater risks, including resources scarcity and habitat fragmentation. In rugged terrain and dense vegetation like Jangsudae, older individuals can secure enough cover and resources, while younger or less experienced gorals might struggle to survive and influence the population’s age and sex structure. Supporting this, Kim et al. (2018) found environmental factors to be a major cause of goral mortality, with males being disproportionately affected. The survey period (July–October) did not include the peak rutting season of long-tailed goral, during which males are more active and detectable and this seasonal exclusion may have contributed to the lower number of males recorded.

The observed patterns may also be influenced by additional ecological factors. Habitat fragmentation, as discussed by Mullu (2016), can isolate individuals, especially males, because of their larger home ranges and exploratory behaviour. Additionally, competition amongst males for resources during harsh seasons might exacerbate their vulnerability. To understand these dynamics, more research is needed including direct monitoring of mortality causes, behavioural study and long-term population dynamics across different habitats. Such research will provide more insight into the mechanisms of sex ratio imbalance in goral population and provide information for more effective conservation strategy.

This study provides valuable information on the population structure and demographics of long-tailed gorals in Jangsudae. However, its relatively small survey area (5.76 km^2^) may not fully represent the entire goral population of the region. Additionally, while camera traps are effective for population assessments, their effectiveness can be limited by dense vegetation and rugged terrain, which may hinder consistent data collection on age and sex distribution, particularly for younger or less mobile individuals. Furthermore, the use of camera traps may generate biases due to variations in detection rates and difficulties in differentiating individuals in overlapping or low-resolution images.

## Conclusions

Our study offers valuable insights into the population structure, age distribution and sex ratio of long-tailed gorals in the Jangsudae Region of Seoraksan National Park, Republic of Korea. Using camera traps, we identified 29 individual gorals and observed a mature age structure, with female biased sex ratio and limited representation of young individuals. This study helps us to understand the ecological requirements of long-tailed gorals and provides a foundation for conservation planning in Republic of Korea and other regions with similar conservation issues. Future studies should focus on relatively broad surveys and other data collection methods to better understand the dynamics of goral population.

## Figures and Tables

**Table 1.** Detailed information on the 29 individual gorals in Jangsudae, identified through morphological classification of images captured by camera traps.

Individual ID	Code	Sex	Individual ID	Code	Sex
SJ-01	A-2-a	Male	SJ-16	A-1-a	Female
SJ-02	A-1-c	Female	SJ-17	A-1-d	Female
SJ-03	A-1-d	Female	SJ-18	B-N	-
SJ-04	A-2-b	Male	SJ-19	A-1-a	Female
SJ-05	A-5-c	Female	SJ-20	B-N	-
SJ-06	A-1-d	Female	SJ-21	A-3-b	Female
SJ-07	A-2-a	Male	SJ-22	A-5-b	Male
SJ-08	A-1-a	Female	SJ-23	A-7-b	Female
SJ-09	B-N	-	SJ-24	A-7-a	Female
SJ-10	A-5-b	Female	SJ-25	A-1-c	Female
SJ-11	A-7-a	Male	SJ-26	A---	Female
SJ-12	A-3-c	Female	SJ-27	B-N	-
SJ-13	B-N	-	SJ-28	B-Y	-
SJ-14	A-2-a	Male	SJ-29	A-2-d	Female
SJ-15	A-1-a	Male			
